# Evolutionary analysis of apolipoprotein E by Maximum Likelihood and complex network methods

**DOI:** 10.1590/1678-4685-GMB-2015-0164

**Published:** 2016-07-14

**Authors:** Leandro de Jesus Benevides, Daniel Santana de Carvalho, Roberto Fernandes Silva Andrade, Gilberto Cafezeiro Bomfim, Flora Maria de Campos Fernandes

**Affiliations:** 1 Universidade Federal de Minas Gerais (UFMG), Belo Horizonte, MG, Brazil; 2 Universidade Federal da Bahia (UFBA), Salvador, BA, Brazil

**Keywords:** Apolipoprotein E, phylogeny, complex network, Maximum Likelihood

## Abstract

Apolipoprotein E (apo E) is a human glycoprotein with 299 amino acids, and it is a major component of very low density lipoproteins (VLDL) and a group of high-density lipoproteins (HDL). Phylogenetic studies are important to clarify how various apo E proteins are related in groups of organisms and whether they evolved from a common ancestor. Here, we aimed at performing a phylogenetic study on apo E carrying organisms. We employed a classical and robust method, such as Maximum Likelihood (ML), and compared the results using a more recent approach based on complex networks. Thirty-two apo E amino acid sequences were downloaded from NCBI. A clear separation could be observed among three major groups: mammals, fish and amphibians. The results obtained from ML method, as well as from the constructed networks showed two different groups: one with mammals only (C1) and another with fish (C2), and a single node with the single sequence available for an amphibian. The accordance in results from the different methods shows that the complex networks approach is effective in phylogenetic studies. Furthermore, our results revealed the conservation of apo E among animal groups.

## Introduction

Apolipoprotein E (apo E) is a human glycoprotein composed of 299 aminoacids (Wernette-Hammonds *et al.*, 1989). It is one of the main proteins in plasma, to where it is exported after being synthesized, mainly in the liver (Elshourbagy and Walker, 1986; Lin *et al.*, 1986). In the cytoplasm, apo E is the major component of very low density lipoproteins (VLDL). It is also part of a high-density lipoproteins group (HDL) (Mensenkamp *et al.*, 1999) that play a key role in triglyceride-rich component catabolism in humans (Kasap *et al.*, 2008). Apo E deficiencies cause diseases related to the increase in cholesterol and triglycerides level in the circulation, such as hyperlipidemia, coronary heart disease, Alzheimer's disease, and other neurodegenerative diseases (Lohse *et al.*, 1991; Corder *et al.*, 1993; Mahley and Huang 1999).

There are other apolipoproteins related to triglyceride and cholesterol redistribution in animals, and depending on the organism, different lipoprotein transport systems can exist using similar mechanisms (Davis, 1997). For instance, apo E is not found in birds, but a homologous apolipoprotein (apo A-I) performs functions that are similar to those of apo E in humans and other vertebrates (Rajavashisth *et al.*, 1987; Lamon-Fava *et al.*, 1992). Hence, to understand the role of apo E within groups of organisms, phylogenetic studies may contribute to elucidate questions about how apo E proteins are related in different vertebrates, and whether they may have evolved from a common ancestor (Kasap *et al.*, 2008).

Currently, phylogenetic results are continuously improved due to the increasing availability of a large amount of biological data and to new approaches to analyze them. Regarding the number of organisms included in a previous study (Kasap *et al.*, 2008), it is now possible to obtain a phylogeny based on more than twice as many organisms as before and with different methodologies. Here, we first conducted a phylogenetic reconstruction based on the Maximum Likelihood (ML) method, which is one of the most reliable ones among the available methods. ML is a phylogenetic inference method that uses probabilistic models for representing the tree with the highest probability or likelihood of correctly reproducing the observed data. The ML criterion allows the analysis of several variables, which makes the method robust, but with increasing computational cost. Another method for phylogenetic reconstruction that is also robust and demands less computational cost, is Bayesian Inference (BI). In practice, ML and BI should present similar and accurate results, but we decided not to use BI because we did not have a problem with the computational cost for our data and because there is some controversy about this method. For example, some studies suggest that BI can be more prone to long-branch attraction biases than ML techniques, and that there could be some problems with Markov chain Monte Carlo (MCMC) implementations or prior distributions (Kolaczkowski and Thornton, 2009). In addition to the ML method, several complex network-based approaches have been proposed, which allow for the analysis of larger datasets with relatively low computational cost (Andrade *et al.*, 2011). Such methods have been widely applied to biological evolutionary studies (Junker and Schreiber, 2008; Boccaletti, 2009).

Therefore, a second set of results for a complex network-based phylogenetic classification is presented in this study, which uses the network modularity concept. It uses the apo E similarity scores evaluated for each pair of organisms in the considered set to identify network communities (or modules). Here, the nodes of each community have a larger number of connections with other nodes within the same modules, in comparison to the connections they have with nodes outside of it. The phylogenetic tree then results from the identification of modules. Note that when the nodes have connections within their own modules, the respective communities become isolated.

Summarizing, this study aimed at performing a phylogenetic analysis based on a set of 32 apo E amino acid sequences using both the ML method and a reliable complex network based approach (Andrade *et al.*, 2011). The latter does not require further biological assumptions for its application, except for the ones used by BLAST to calculate similarity scores. As it will become clear in the next sections, the results obtained using the two different approaches are in conformity and helped to clarify how various apo E proteins are related among different groups of animals.

## Material and Methods

### Sequences

The apo E sequences were downloaded from NCBI (National Center for Biotechnology Information, http://www.ncbi.nih.gov) on November 17, 2014 (Table S1). Based on homologous sequences deposited in the HomoloGene database (http://www.ncbi.nlm.nih.gov/homologene), two BLAST (Basic Local Alignment Search Tool) analyses (Altschul *et al.*, 1997) have been performed: the first one used the *Homo sapiens* apo E sequence as query, whereas in the second one the *Danio rerio* apo E sequence was the query used. Only sequences with an E-value lower than e10^−6^ and without the RefSeq status PREDICTED were considered. The only representative amphibian sequence (*Xenopus (Silurana) tropicalis*) found in NCBI was the same as the one used by Kasap *et al.* (2008). Once collected, the sequences were aligned computationally using ClustalW (Thompson *et al.*, 1994) default parameters. The alignment was subsequently revised and manually edited in BioEdit 7.1.3.0 (Hall 1999), following the instructions in Hall (2011).

### Maximum Likelihood

After alignment and editing, the dataset was first subjected to the ML phylogenetic analysis. The MEGA6 software (Tamura *et al.*, 2013) was used to select the best evolutionary substitution model of amino acid residues for this dataset, whereas the Tree-Puzzle 5.2 software (Schmidt *et al.*, 2002) was used for likelihood mapping analysis. To perform the phylogenetic reconstruction, MEGA6 was used under the ML criterion with 1000 bootstrap replicates.

### Complex Networks

In the first step of the complex network analysis, a local pairwise alignment was performed using BLAST version 2.2.21 (StandAlone), which resulted in a similarity *S* matrix with elements *0*≤*s*_*i,j*_≤*100* consisting of the mean value of the pairwise similarity scores between sequences (*i,j*) and (*j,i*). The similarity values were considered valid for those pairs of sequences with an E-value equal to or smaller than 1.0. Next, the community identification started with the construction of the one-parameter network family, as proposed by Andrade *et al.* (2011).

The symmetric *S* matrix allows the construction of a one-parameter family of undirected networks, which is generated according to the following scheme: i) the apo E sequences were considered as nodes; ii) the edges, representing interactions between nodes, depend on the similarity degree of the sequences; iii) a control parameter, represented by a threshold similarity *S_th_*=*σ*, allows generating a network family of adjacency matrices *M(σ)*, such that the matrix elements *M_ij_(σ)* can only assume the values 0 or 1; iv) these values indicate, respectively, the absence or presence of an edge between nodes *i* and according to the following prescription: *M_ij_(σ)*=1 *if s_ij_*3 *σ*; *M_ij_(σ)*=*0 if s_ij_* < *σ*.

Finally, the modularity is obtained by two properties of the network family. The first one is the network distance δ between two networks evaluated at nearby σ values, denoted by δ(σ,σ+Δσ). The graph of δ as function of σ allows identifying the optimal σ_*cst*_ values at which the network undergoes significant structural changes that can be associated with the emergence of communities separated from the rest of the network. The critical networks graphical representation was done through GePhi software (Bastian *et al.*, 2009). The phylogenetic classification is completed by the construction of a dendrogram with the σ value representing the horizontal axis, in which communities or isolated nodes give rise to subtrees or individual branches at the σ-values where they become disconnected from the other modules or completely isolated from any other node. It is worthy of note that the construction of the dendrogram in the current study relied only on community separation by changing the σ value. This methodological change could be made because the data set in the current work was much smaller than those data sets used in previous studies (Góes-Neto *et al.*, 2010; Andrade *et al.*, 2011), in which the dendrograms at fixed σ values were obtained by successive link elimination based on Newman-Girvan's algorithm. Nonetheless, we also obtained the same results reported in the next section by applying of the previously two-step procedure.

## Results

### Phylogeny

**Figure 1:**
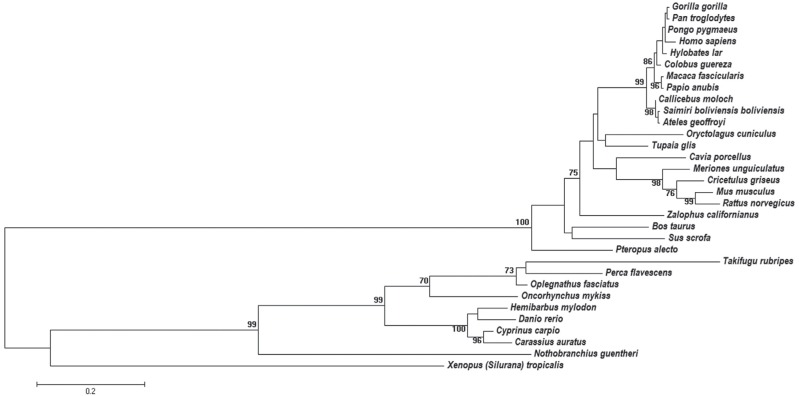
Phylogenetic tree of apo E sequences constructed by the ML method. The bootstrap analysis was performed with 1000 pseudoreplicates. Bootstrap values lower than 70% are not shown. The evolutionary distances were computed using the Jones-Taylor-Thornton (JTT) model.

The obtained dataset was composed of 32 apo E amino acid sequences. Data mining of the NCBI records did not detect apo E sequences of birds and reptiles. The multiple alignments of these sequences, after manual editing, contained 250 residues. The analysis based on the Likelihood Ratio Test indicated that the evolutionary substitution model of amino acid residues that better explains the data is the Jones-Taylor-Thornton one (JTT) (Jones *et al.*, 1992), with *gamma* distribution (α *= 3.8665*). The likelihood mapping result indicated the presence of a phylogenetic signal with 88.8% of quartets located at the vertices and 7.7% in the center-dimensional simplex. The unrooted phylogenetic tree is shown in Figure 1, as a phylogram.

### Complex Network

**Figure 2:**
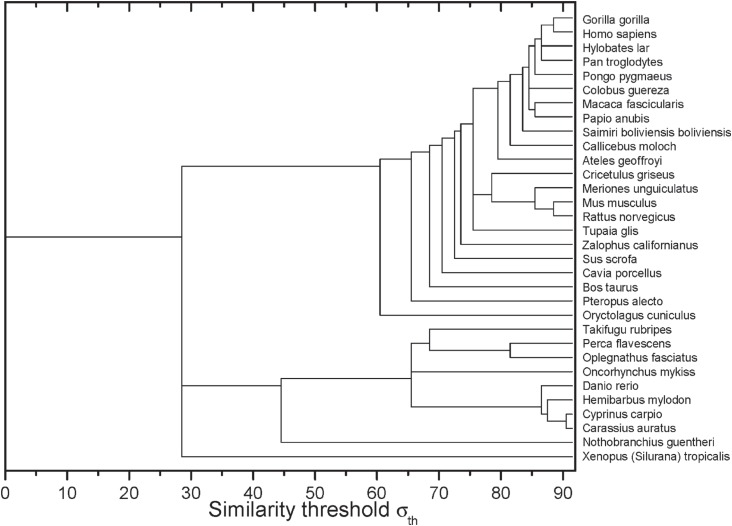
Complex networks analysis dendrogram showing the edge elimination dynamics according to the similarity threshold σ (in %).

After the construction of networks at different σ values, it was possible to obtain the dynamics of edges elimination represented by the dendrogram shown in Figure 2. In the horizontal axis, it is possible to identify the σ−values in which the sequences of two or more organisms have lost their connection with the other sequences.

**Figure 3:**
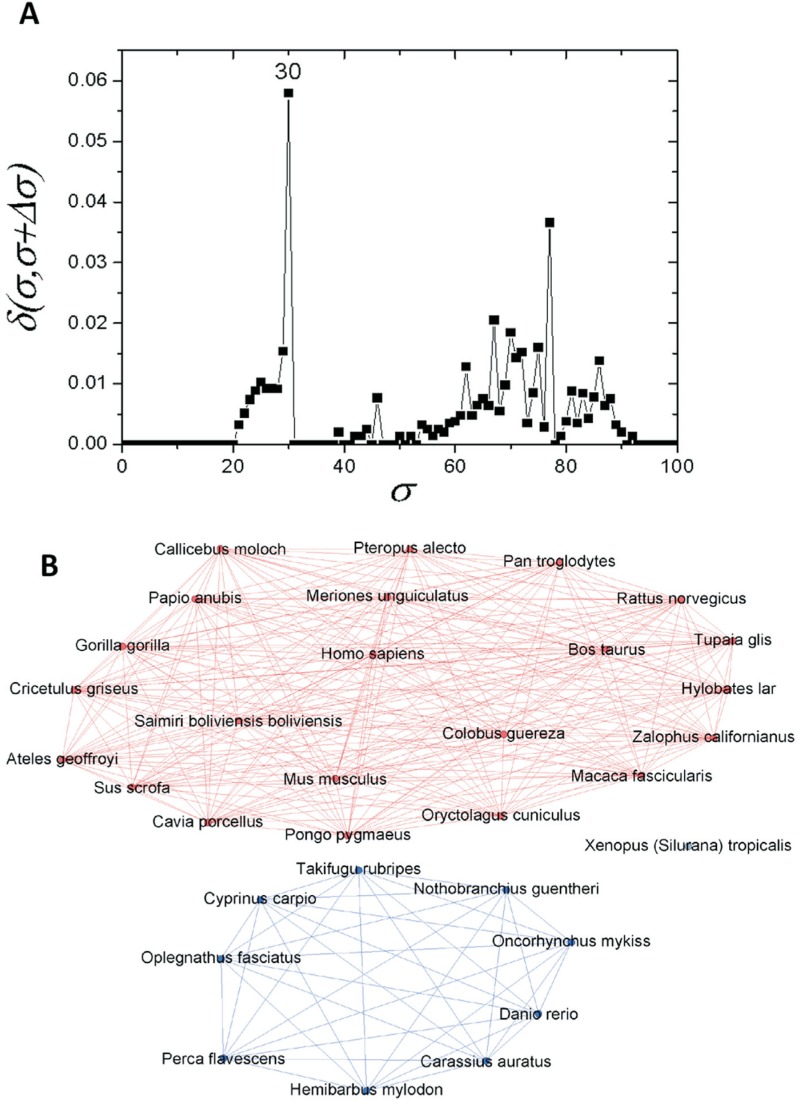
Complex networks analysis. (A) Graph showing the δ-distance between the networks, emphasizing the indication of the branching event at σ*_cst_*=*30%*. Smaller peaks related to branching events in primate and fish sub-communities are highlighted in Figures 5A and 6A. (B) Representation of apo E network at σ*_cst_*=*30%.*

These branching events are clearly revealed by the δ-distance graph shown in Figure 3A. It shows that the apo E network family undergoes the most important structural change at σ=*30%*, switching from a completely connected graph to a modular structure consisting of two communities (C) and an isolated node (Figure 3B). They correspond, respectively, to two communities of organisms in the databases [mammalian (C1) and fish (C2)], while the sequence corresponding to frog remained isolated (Figure 3B).

**Figure 4:**
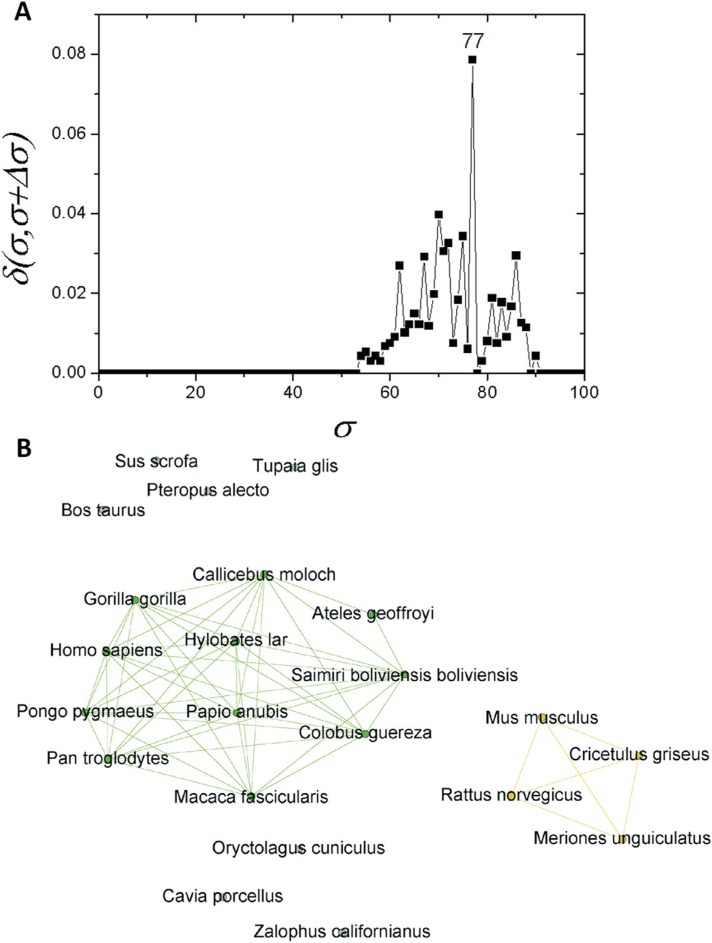
Complex networks analysis. (A) Graph showing the δ-distance between networks formed by the apo E sequences from mammals with σ*_cst_*=*77%*. (B) Network representation of apo E mammal sequences at σ*_cst_*=*77%*.

**Figure 5:**
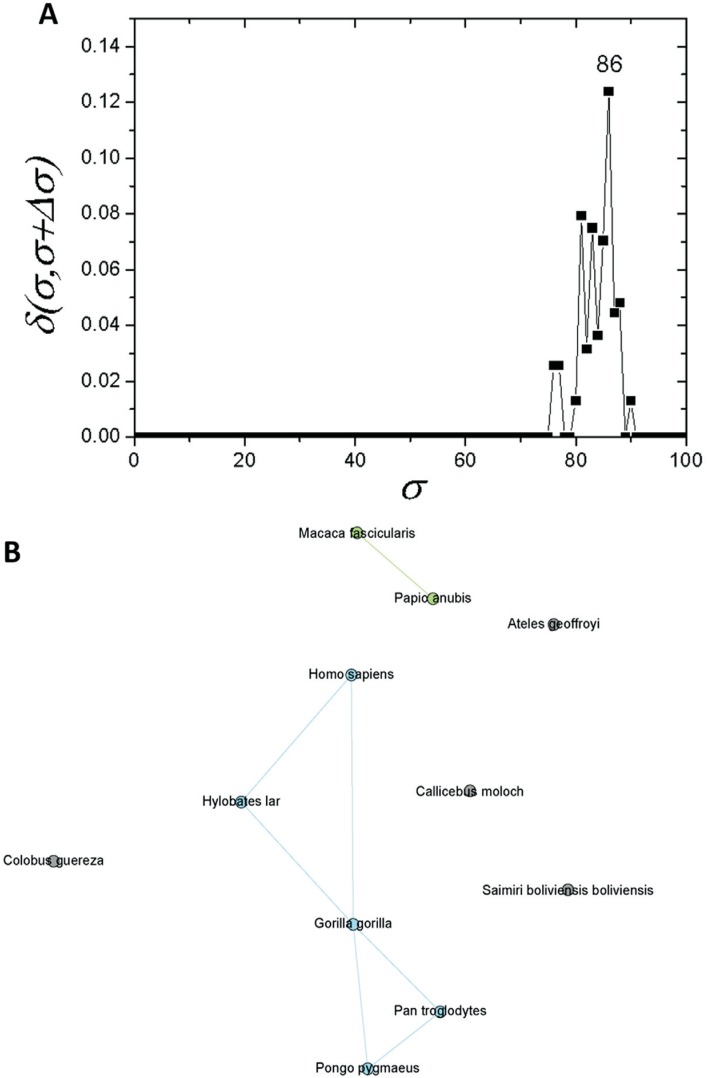
Complex networks analysis. (A) Graph showing the δ-distance between networks formed by the apo E sequences from primates with σ*_cst_*=*86%*. (B) Network representation of apo E primate sequences at σ*_cst_*=*86%*.

If one considers a σ > *30%*, the C1 community can be regarded as an independent network composed of mammals only. If a similar structure identification based on δ-distance is carried out, a new critical threshold value of σ*_cst_*=*77%* is obtained (Figure 4A). The network obtained with this σ_*cst*_-value separated two communities, one composed of primates (C1.1) and the other of four rodents (C1.2). Prior to this, seven further sequences were separated: *T. glis, O. cuniculus, S. scrofa, P. alecto, B. taurus, C. porcellus* and *Z. californianus* (Figure 4B). As C1 revealed a subcommunity with 11 primates (C1.1), an analysis was performed for this particular subcommunity. It was possible to detected a new branching event at σ_*cst*_=*86%* (Figure 5A). Among the species within C1.1, five sequences (*G. gorilla, H. sapiens, P. pygmaeus, P. troglodytes,* and *H. lar*) were the most interconnected ones (Figure 5B).

**Figure 6:**
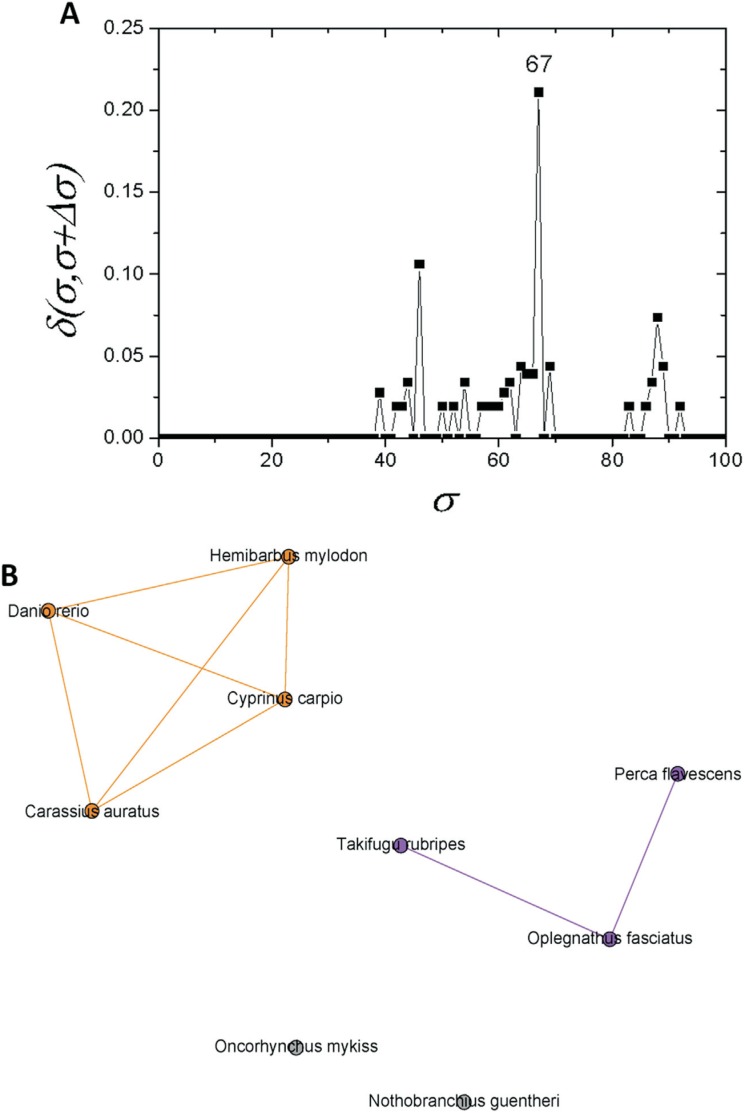
Complex networks analysis. (A) Graph showing the δ-distance between networks formed by the apo E sequences from fishes with maximum δ values at σ= 67. (B) Network representation of apo E fish sequences at σ*_cst_*=*67%*.

The analysis of community C2, which contains apo E sequences from fish, led to the identification of three further significant σ_*cst*_-values, at 46%, 67% and 88%, as shown in Figure 6A. A fish sub-network constructed for σ=*46%* showed that only the *N. guentheri* sequence separates from the others, whereas in the network obtained for σ=*67%*, the *O. mykiss* apo E sequence was also separated from two other subcommunities: C2.1 (*T. rubripes, O. fasciatus* and *P. flavescens*) and C2.2 (*Danio rerio, H. mylodon, C. auratus* and *C. carpio*) (Figure 6B). Finally, at σ=*88%* in C2.2, the *D. rerio* sequence was separated from the others.

## Discussion

This study considered 32 apo E amino acid sequences deposited in the NCBI database. Apo E sequences from birds and reptiles were not found in the search, which is in accordance with previous studies suggesting that apo E does not exist in some groups, and that, probably, other apolipoprotein homologues perform a corresponding function (Lamon-Fava *et al.*, 1992; Duggan and Callard, 2001).

The construction of a complex network family was based on the method proposed by Andrade *et al.* (2011). It allows a comparison between two networks obtained at close similarity thresholds, using the concept of δ-distance between complex networks (Andrade *et al.*, 2008). Thus, changes in the network structure can be detected just by drawing a graph of the δ-distance between networks as function of the similarity threshold value of σ. This procedure allows identifying a few critical similarity threshold values of σ_*cst*_, at which an optimal network can be generated with clear-cut modular properties.

The phylogenetic tree obtained by ML is not rooted because there was no sequence included that could be used as outgroup. In this tree, a clear separation can be observed between three classes: Mammalia, Amphibia and Actinopterygii (Figure 1). The branch representing the class Mammalia is supported by bootstrap score of 100, whereas the one representing the Actinopterygii shows bootstrap score of 99. The branches having bootstrap scores above 70 can be considered as well supported in phylogenetic trees constructed from amino acid sequence data. Hence, the branches representing Mammalia and Actinopterygii in this study are well supported.

These results were corroborated by the use of a complex network method, which indicated a first splitting of the original full connected organism group into two different communities and an isolated node. The first community contained only mammals (C1), the second one was composed of fish (C2), while the amphibian sequence (*X. tropicalis*) became detached from all other nodes (Figure 3B). Due to the low σ_*cst*_-value necessary to separate these communities (30%) (Figure 3A), it is possible to infer that the conservation of apo E sequences among the different metazoan classes is very low.

Moreover, a much higher apo E conservation could be observed within the Mammalia class. This result is in accordance with the phylogenetic tree shown in Figure 1, in which the class Mammalia was supported with a bootstrap value of 100, and in the network shown in Figure 4, in which it was possible only at σ_*cst*_ = *77%* to observe a significant new separation of the sequences.

Other well-supported clusters shown in Figure 1 are the orders Rodentia (*M. musculus* and *R. norvegicus*) (bootstrap 99) and Primates (bootstrap 99). The topology of the tree shown in Figure 1 is in accordance with the proposed phylogenetic tree for vertebrates in the Tree of Life Web Project (Janvier, 1997).

Within the group of primates a polytomy was found, which encompasses four taxa of the Hominidae family (Figure 1). It was likely not resolved by the ML method due to the high degree of apo E sequence conservation in these primates. This high degree of conservation was also seen in the complex networks analysis (Figure 2). At σ*_cst_*=*84%*, the last grouped sequences (*H. sapiens, P. troglodytes, H. lar, P. pygmaeus* and *G. gorilla*) are split from the other primates. Considering the Hominidae clade, *P. pygmaeus* separated from the other four sequences at σ_*cst*_ =*85%*, while for *Pan troglodytes* and *Hylobates lar* the separation occurred at σ_*cst*_ =*86%*. Finally, *H. sapiens* and *G. gorilla* separated from each other at σ_*cst*_ =*88%* (Figure 2). Although the joint separation of taxa may occur at the same threshold, it does not necessarily indicate that these taxa belong to the same clade. It probably occurs due to the high degree of apo E conservation, as pointed out above. In addition, it is interesting to note that *H. sapiens* and *G. gorilla* remain connected until σ_*cst*_ = 88%, confirming the proximity between *Pan, Homo* and *Gorilla*, which is in accordance with the literature (see, *e.g.*, Perelman *et al.*, 2011). Thus, the complex network method used proved to be reliable and sensitive to small changes in sequence similarity, since it was able to detect the expected communities based only on the apo E sequences from these primates.

The network composed only by primate sequences (Figure 5A) for σ*_cst_*=*86%* supports the conservation of apo E. The Hominidae apoE sequences were placed in the same branch, and they are connected in the network analysis (Figure 5B). Hence, it confirms high protein conservation, and the challenge of obtaining a fully resolved phylogeny, even with assistance of the complex network analyses.

Among fish, represented in this study by the class Actinopterygii, the tree is fully resolved and apo E conservation within the orders is once again evident. This is denoted by the well supported branch (bootstrap 100) that represents the family *Cyprinidae*.

For σ_*cst*_=67%, the network analysis performed only with fish apo E sequences indicated the presence of two groups: the first one formed by the family *Cyprinidae* and the other by two species from the order *Perciformes* (*Oplegnathus fasciatus* and *Perca flavescens*), and one species from the order *Tetraodontiformes* (*Takifugu rubripes*). Notably, the results from the two methods used in this study were similar. Given the huge amount of data available, our results also indicated that the development of new approaches, like those based on complex network theory, can be important to support and advance phylogenetic studies, as already suggested by Goes-Neto *et al.* (2010) and Andrade *et al.* (2011).

From an evolutionary point of view it is interesting to note that the similarity threshold between fish, amphibians and mammals is low (30%, Figure 3A). Noting that lipoproteins can play an important role in dietary function, this result was expected and as seen in other studies (Finch and Stanford, 2004; Kasap *et al.*, 2008; McIntosh *et al.*, 2012), the diet from different animal groups is very divergent, shaping Apo E differently in these animals. Despite the clear divergence between mammals, fishes and amphibians, our methods were able to detect significant divergence between apo E within these groups, as seen in primates and rodents, for example. Furthermore, our results suggest that this lipoprotein presents an adaptive flexibility with regard to the separation of the vertebrate groups studied here. Thus, from the low similarity percentage needed to separate different organism classes (Amphibia, Chondrichthyes and Mammalia), we infer that apo E was under strong selective pressure in each of the classes, possibly because of dietary divergence. Dietary divergence may have lead to significant changes in apo E so that it was possible to see a clear distance in protein similarity between orders (*e.g.* Rodentia, Primates).

## Supplementary material

The following online material is available for this article:

Table S1 - GenBank access numbers for apo E sequences.
