# Supplementary material for: Evolutionary analysis of apolipoprotein E by Maximum Likelihood and complex network methods
Source: Genet Mol Biol. 2016 Jul 14;39(4):665–73. doi: 10.1590/1678-4685-GMB-2015-0164 (PMC5127143; doi:10.1590/1678-4685-GMB-2015-0164)
Supplement: Supplementary file 1 [file 1415-4757-gmb-1678-4685-GMB-2015-0164-Suppl01.pdf]

Table S1 - GenBank access numbers for apo E sequences.

Organisms	Access numbers	Autors
Ateles geoffroyi (black-handed spider monkey)	P0DKW6.1	Cheng,J.-F., Hamilton,M., Peng,Y., Hosseini,R., Peng,Z., Malinov,I. and Rubin,E.M.
Bos taurus (cattle)	NP_776416.1	Zimin AV, Delcher AL, Florea L, Kelley DR, Schatz MC, Puiu D, Hanrahan F, Pertea G, Van Tassell CP, Sonstegard TS, Marçais G, Roberts M, Subramanian P,Yorke JA, Salzberg SL.
Callicebus moloch (Red-bellied titi)	P0DKW7.1	Cheng,J.-F., Hamilton,M., Peng,Y., Hosseini,R., Peng,Z., Malinov,I. and Rubin,E.M.
Carassius auratus (goldfish)	AEX91933.1	Wang,F. and Liu,S.
Cavia porcellus (domestic guinea pig)	P23529.1	Matsushima,T., Getz,G.S. and Meredith,S.C.
Colobus guereza (mantled guereza)	P0DKU9.1	Cheng,J.-F., Hamilton,M., Peng,Y., Hosseini,R., Peng,Z., Malinov,I. and Rubin,E.M.
Cricetulus griseus (Chinese hamster)	ERE59408.1	Brinkrolf,K., Rupp,O., Laux,H., Kollin,F., Ernst,W., Linke,B.,Kofler,R., Romand,S., Hesse,F., Budach,W.E., Galosy,S., Muller,D.,Noll,T., Wienberg,J., Jostock,T., Leonard,M., Tauch,A., Goesmann,A., Helk,B., Mott,J.E., Puehler,A. and Borth,N.Grillari,J.
Cyprinus carpio (common carp)	AEX91934.1	Wang,F. and Liu,S.
Danio rerio (zebrafish)	NP_571173.1	Shiau CE, Monk KR, Joo W and Talbot WS.

Gorilla gorilla (western gorilla)	AAG28579.1	Rogaev,E.I., Dvorianchikov,G.A. and Riazanskaia,N.N.
Hemibarbus mylodon (Korean doty barbel)	ACI15893.1	Kim,K.Y., Cho,Y.S., Bang,I.C. and Nam,Y.K.
Homo sapiens (human)	NP_000032.1	Poursadegh Zonouzi A, Farajzadeh D, Bargahi N and Farajzadeh M.
Hylobates lar (common gibbon)	AAG28581.1	Rogaev,E.I., Dvorianchikov,G.A. and Riazanskaia,N.N.
Macaca fascicularis (crab-eating macaque)	CAA32092.1	Marotti,K.R., Whitted,B.E., Castle,C.K., Polites,H.G. and Melchior,G.W.
Meriones unguiculatus (Mongolian gerbil)	ADV16116.1	Liu,Y. and Wu,J.
Mus musculus (house mouse)	NP_033826.2	Du F, Yu F, Wang Y, Hui Y, Carnevale K, Fu M, Lu H and Fan D.
Nothobranchius guentheri (redtail notho)	AEO44886.1	Shang,X.M.
Oncorhynchus mykiss (rainbow trout)	CAB65320.1	Durliat,M., Andre,M. and Babin,P.J.
Oplegnathus fasciatus (barred knifejaw)	ACF21982.1	Kim,K.-Y., Cho,Y.S., Kim,S.K. and Nam,Y.K.
Oryctolagus cuniculus (rabbit)	NP_001076112.1	Carlucci A, Cigliano L, Maresca B, Spagnuolo MS, Di Salvo G,Calabro R and Abrescia P.
Pan troglodytes (chimpanzee)	NP_001009007.1	McIntosh AM, Bennett C, Dickson D, Anestis SF, Watts DP, Webster TH, Fontenot MB and Bradley BJ.
Papio anubis (Olive baboon)	AAA35381.1	Hixson,J.E., Cox,L.A. and Borenstein,S.
Perca flavescens (yellow perch)	ACO36146.1	
Pongo pygmaeus (Bornean orangutan)	AAG28580.1	Rogaev,E.I., Dvorianchikov,G.A. and Riazanskaia,N.N.
Pteropus alecto (black flying fox)	ELK12734.1	Zhang,G., Cowled,C., Shi,Z., Huang,Z., Bishop-Lilly,K.A., Fang,X.,Wynne,J.W., Xiong,Z., Baker,M.L., Zhao,W.,

		Tachedjian,M., Zhu,Y.,Zhou,P., Jiang,X., Ng,J., Yang,L., Wu,L., Xiao,J., Feng,Y.,Chen,Y., Sun,X., Zhang,Y., Marsh,G.A., Crameri,G., Broder,C.C.,Frey,K. G., Wang,L.F. and Wang,J.
Rattus norvegicus (Norway rat)	NP_001257610.1	Tran TN, Kim SH, Gallo C, Amaya M, Kyees J and Narayanaswami V.
Saimiri boliviensis boliviensis (Bolivian squirrel monkey)	P0DKW8.1	Cheng,J.-F., Hamilton,M., Peng,Y., Hosseini,R., Peng,Z., Malinov,I. and Rubin,E.M.
Sus scrofa (pig)	NP_999473.1	Li S, Zhang H, Gao P, Chen Z, Wang C and Li J.
Takifugu rubripes (Fugu rubripes)	NP_001072103.1	Kondo,H., Morinaga,K., Misaki,R., Nakaya,M. and Watabe,S.
Tupaia glis (common tree shrew)	AAG21401.1	Zhang,J., Chen,B., Zeng,W., Wu,G., Zhang,W., Fang,L. and Xue,H.
Xenopus (*Silurana*) *tropicalis* (western clawed frog)	AAH75258.1	Klein,S.L., Strausberg,R.L., Wagner,L., Pontius,J., Clifton,S.W. and Richardson,P.
		Klein,S.L., Strausberg,R.L., Wagner,L., Pontius,J., Clifton,S.W. and Richardson,P.
Zalophus californianus (California sea lion)	Q7M2U7.1	Davis,R.W., Pierotti,V.R., Lauer,S.J., Hubl,S.T., McLean,J.W., Witztum,J.L. and Young,S.G.
